# T222. EARLY TREATMENT RESISTANCE IN A LATIN-AMERICAN COHORT OF PATIENTS WITH SCHIZOPHRENIA

**DOI:** 10.1093/schbul/sby016.498

**Published:** 2018-04-01

**Authors:** Cristian Mena, Alfonso Gonzalez-Valderrama, Barbara Iruretagoyena, Juan Undurraga, Nicolas Crossley

**Affiliations:** 1 J. Horwitz Psychiatric Institute; 2 Universidad Católica de Chile

## Abstract

**Background:**

Failure to respond to antipsychotic medication in schizophrenia is a common clinical scenario with significant morbidity. Recent studies have highlighted that many patients present treatment-resistance from disease onset. We here present an analysis of clozapine prescription patterns, used as a real-world proxy marker for treatment-resistance, in a cohort of 1195patients with schizophrenia from a Latin-American cohort, to explore the timing of treatment resistance during the course of the disease and possible subgroup differences.

**Methods:**

We used survival analysis from national databases of clozapine monitoring system, national disease notification registers, and discharges from an early intervention ward.

**Results:**

Echoing previous studies, we found that around 1 in 5 patients diagnosed with schizophrenia were eventually prescribed clozapine, with an over-representation of males and those with a younger onset of psychosis. The annual probability of being prescribed clozapine was highest within the first year (probability of 0.11, 95% confidence interval of 0.093–0.13), compared to 0.018 (0.012–0.024) between years 1 and 5, and 0.006 (0–0.019) after 5 years. There were no differences in age at psychosis onset or gender related to the onset of treatment resistance. A similar pattern was observed in a subgroup of 230 patients discharged from an early intervention ward with a diagnosis of non-affective first episode of psychosis.

**Discussion:**

Our results highlight that treatment resistance is frequently present from the onset of psychosis. Future studies will shed light on the possible different clinical and neurobiological characteristics of this subtype of psychosis.

